# Loop-Mediated Isothermal Amplification (LAMP) Method for Rapid Detection of *Trypanosoma brucei rhodesiense*

**DOI:** 10.1371/journal.pntd.0000147

**Published:** 2008-02-06

**Authors:** Zablon Kithinji Njiru, Andrew Stanislaw John Mikosza, Tanya Armstrong, John Charles Enyaru, Joseph Mathu Ndung'u, Andrew Richard Christopher Thompson

**Affiliations:** 1 School of Nursing, Murdoch University, Mandurah, Western Australia, Australia; 2 Trypanosomiasis Research Centre, Kenya Agricultural Research Institute, Kikuyu, Kenya; 3 WHO Collaborating Centre for the Molecular Epidemiology of Parasitic Infections, School of Veterinary and Biomedical Sciences, Murdoch University, Murdoch, Western Australia, Australia; 4 Department of Biochemistry, Faculty of Science, Makerere University, Kampala, Uganda; 5 Foundation for Innovative New Diagnostics (FIND), Cointrin, Switzerland; Yale University School of Medicine, United States of America

## Abstract

Loop-mediated isothermal amplification (LAMP) of DNA is a novel technique that rapidly amplifies target DNA under isothermal conditions. In the present study, a LAMP test was designed from the *serum resistance-associated* (*SRA*) gene of *Trypanosoma brucei rhodesiense*, the cause of the acute form of African sleeping sickness, and used to detect parasite DNA from processed and heat-treated infected blood samples. The *SRA* gene is specific to *T. b. rhodesiense* and has been shown to confer resistance to lysis by normal human serum. The assay was performed at 62°C for 1 h, using six primers that recognised eight targets. The template was varying concentrations of trypanosome DNA and supernatant from heat-treated infected blood samples. The resulting amplicons were detected using SYTO-9 fluorescence dye in a real-time thermocycler, visual observation after the addition of SYBR Green I, and gel electrophoresis. DNA amplification was detected within 35 min. The *SRA* LAMP test had an unequivocal detection limit of one pg of purified DNA (equivalent to 10 trypanosomes/ml) and 0.1 pg (1 trypanosome/ml) using heat-treated buffy coat, while the detection limit for conventional *SRA* PCR was ∼1,000 trypanosomes/ml. The expected LAMP amplicon was confirmed through restriction enzyme *Rsa*I digestion, identical melt curves, and sequence analysis. The reproducibility of the *SRA* LAMP assay using water bath and heat-processed template, and the ease in results readout show great potential for the diagnosis of *T. b. rhodesiense* in endemic regions.

## Author Summary

Control of human African trypanosomiasis (HAT) or sleeping sickness relies on diagnosis and treatment of infected patients. However, the diagnostic tests in routine use have limited sensitivity, due to a characteristically low parasitaemia in infected individuals. Differentiation of infections by *Trypanosoma brucei rhodesiense* (causes acute disease) and *T. b. gambiense* (causes chronic disease) is essential, as the two forms of disease have different treatment regimens. In the present work, loop-mediated isothermal amplification (LAMP) of DNA was successfully used to detect *T. b. rhodesiense,* with a sensitivity of up to one trypanosome/ml of blood. The LAMP test was efficient and robust, and results were obtained within 35 min. Amplification was possible when a water bath was used to maintain the temperature at isothermal conditions (60–65°C), and results could be read by visual observation of colour change. These findings have increased the prospects for developing a simple molecular test for HAT that can be used with limited equipment at point of care in endemic rural areas.

## Introduction

Human African trypanosomiasis is endemic in tropical Africa. In eastern and southern Africa the disease is caused by *Trypanosoma brucei rhodesiense,* while *T. b. gambiense* infections are common in central and West Africa. *T. b. rhodesiense* causes an acute form of disease, whereas *T. b. gambiense* causes a more chronic form. Moreover, the treatment regimen for the two infections is different, expressing the need for a specific diagnostic test for each trypanosome. The geographical demarcation of *T. b. rhodesiense* and *T. b. gambiense* to a large extent forms the basis of trypanosome identification and treatment. In East Africa the introduction of *T. b. rhodesiense* into the *T. b. gambiense* region is certain to occur due to the closeness of the two disease foci and continuous movement of the livestock-reservoir host for *T. b. rhodesiense*. This prospect further obligates the development of test kits that can differentiate the two parasites. The *serum resistance-associated* (*SRA*) gene [1],[2] is conserved and specific to *T. b. rhodesiense* [3]–[5] and therefore provides unequivocal identification of this parasite. It is a low-copy gene, therefore the polymerase chain reaction (PCR) test is inadequate to amplify this target reliably in clinical samples without recourse to parasite multiplication in mice. Besides, available molecular methods of parasite detection require elaborate precision instruments [3]–[7], which make their use under field conditions unfeasible. There is therefore a need for a simplified method of amplification and product detection that would compliment the available tests and make feasible molecular diagnosis for case detection and confirmation of cure in the regions that are endemic for sleeping sickness.

Recently, a technique called loop-mediated isothermal amplification (LAMP) of DNA has been developed [8]. The technique uses four to six primers that recognise six to eight regions of the target DNA, respectively, in conjunction with the enzyme *Bst* polymerase, which has strand displacement activity. The simultaneous initiation of DNA synthesis by multiple primers makes the technique highly specific. The LAMP test is carried out under isothermal conditions (60–65°C) and produces large amount of DNA [8]. The reaction shows high tolerance to biological products [9], meaning that DNA extraction may not be necessary [10], and the product can be inspected visually by the addition of SYBR Green I [11],[12]. Briefly, LAMP proceeds when the forward inner primer (FIP) anneals to the complementary region (F2c) in the target DNA and initiates the first strand synthesis, and then the outer forward primer (F3) hybridises and displaces the first strand, forming a loop structure at one end [8]. This single-stranded DNA serves as template for backward inner primer (BIP)-initiated DNA synthesis and subsequent outer backward (B3)-primed strand displacement DNA synthesis, leading to the formation of dumbbell-shaped DNA structures [8]. The stem-loop thus formed acts as a template, and subsequently one inner primer hybridises to the loop on the product and initiates the displacement DNA synthesis, forming the original stem loop and a new stem loop that is twice as long [13]. The final products are stem-loop DNAs with several inverted repeats of the target DNA, and cauliflower-like structures bearing multiple loops [8].

A number of LAMP tests to detect parasitic protozoa have been designed and used successfully [14]–[16]. The rapidity, specificity, and simplicity of the technique make it appealing for use in trypanosomiasis-endemic regions. The purpose of the present study was to develop a LAMP test for detection of *T. b. rhodesiense* based on the *SRA* gene and compare it with PCR test that is specific for *T. b. rhodesiense*. Our results indicate that the *SRA* LAMP is sensitive and specific and has the potential to be developed into a field-friendly diagnostic test.

## Materials and Methods

### Ethical clearance

Institutional Ethical Clearance for the collection of human samples had been obtained from the Livestock Health Research Institute (LIRI), Tororo, Uganda, and the Uganda National Council of Science and Technology (UNCST), Kampala, Uganda, which records and regulates all research activities in the country. At Murdoch University, Perth, Australia, the use of mice was approved by Murdoch University Animal Ethics Committee (AEC).

### Preparation of template

The trypanosome DNA samples used in this study are shown in Table 1. The samples which most had been passaged in mice were chosen to ensure a wide geographical representation, different times of isolation, and hosts (Table 1). Six samples designated as JE (three each from blood and cerebrospinal fluid [CSF]) were direct isolates from human hosts. The DNA had been prepared using several methods (see footnotes in Table 1). The samples for studying analytical sensitivity and tolerance of LAMP were obtained from the blood of mice infected with *T. b. rhodesiense* and divided into two portions. The first portion was centrifuged at 3,000 rpm for 10 min and the buffy coat was collected, and the second portion was divided into aliquots of 10 µl. Then each of the two portions was mixed with 40 µl of ultrapure water, boiled for 3 min, and centrifuged at 14,000 *g* for 5 min. Samples of 10–15 µl of supernatant were recovered and stored at −20°C for later use.

**Table 1:** Trypanosome Isolates and Amplification Results

Species/Subspecies	Identification Code	Origin	Year of Isolation	Original Host	Specific PCR Results			*SRA*-LAMP Results		
					TBR^a^	*SRA* Gene^b^	TgsGP^c^	SYBR Green I	Gel	Real Time
*T. b. rhodesiense*	ATCC 30027	Tanganyika	1934	Human	+	+	−	+	+	+
*T. b. rhodesiense*	Gambella II^d^	Ethiopia	1968	Human	+	+	−	+	+	+
*T. b. rhodesiense*	LVH 058^d^	Luangwa Valley, Zambia	1974	Human	+	+	−	+	+	+
*T. b. rhodesiense*	LVH 56^d^	Lambwe Valley, Kenya	1978	Human	+	+	−	+	+	+
*T. b. rhodesiense*	LVH 108^d^	Lambwe Valley, Kenya	1980	Human	+	+	−	+	+	+
*T. b. rhodesiense*	TMRS 010^d^	Kasulu, Tanzania	1991	Human	+	+	−	+	+	+
*T. b. rhodesiense*	TMRS 127^d^	Mpanda, Tanzania	1994	Human	+	+	−	+	+	+
*T. b. rhodesiense*	UTRO 2509^d^	Uganda	N/A	Human	+	+	−	+	+	+
*T. b. rhodesiense*	WB56^d^	Uganda	N/A	Human	+	+	−	+	+	+
*T. b. rhodesiense*	KETRI 2355^d^	Busoga, Uganda	1977	Human	+	+	−	+	+	+
*T. b. rhodesiense*	KETRI 3739^e^	Busia, Kenya	2001	Dog	+	+	−	+	+	+
*T. b. rhodesiense*	KETRI 1900^e^	Lambwe Valley, Kenya	1971	Hyena	+	+	−	+	+	+
*T. b. rhodesiense*	KETRI 2492^e^	Lambwe Valley, Kenya	1980	Tsetse fly	+	+	−	+	+	+
*T. b. rhodesiense*	KETRI 2532^e^	Lambwe Valley, Kenya	1980	Cow	+	+	−	+	+	+
*T. b. rhodesiense*	KETRI 3007^e^	Busia, Kenya	1987	Pig	+	+	−	+	+	+
*T. b. rhodesiense*	JE1	Busoga, Uganda	1990	Human	+	−	−	+	+	+
*T. b. rhodesiense*	JE2^f^	Tororo, Uganda	1991	Human	−	−	−	+	+	+
*T. b. rhodesiense*	JE3^f^	Tororo, Uganda	2005	Human	−	−	−	+	+	+
*T. b. rhodesiense*	JE4^f^	Tororo, Uganda	2002	Human	+	−	−	+	+	+
*T. b. rhodesiense*	JE5	Serere, Uganda	2001	Human	+	+	−	+	+	+
*T. b. rhodesiense*	JE6	Serere, Uganda	2001	Human	+	+	−	+	+	+
*T. b. rhodesiense*	JE8^g^	Tororo, Uganda	2001	Human	−	−	−	+	+	+
*T. b. rhodesiense*	JE9^g^	Tororo, Uganda	2001	Human	+	−	−	+	+	+
*T. b. rhodesiense*	JE10^g^	Tororo, Uganda	2001	Human	+	−	−	+	+	+
*T. b. rhodesiense*	JE12	Serere, Uganda	2003	Human	+	+	−	+	+	+
*T. b. rhodesiense*	JE13	Serere, Uganda	2003	Human	+	+	−	+	+	+
*T. b. rhodesiense*	JE14	Serere, Uganda	2001	Human	+	+	−	+	+	+
*T. b. rhodesiense*	STIB849^h^	Uganda	1991	Human	nd	nd	nd	+	+	+
*T. b. rhodesiense*	AnTat25.1^h^	Rwanda	1971	Human	nd	nd	nd	+	+	+
*T. b. rhodesiense*	AnTat12.1^h^	Rwanda	1991	Human	nd	nd	nd	+	+	+
*T. b. rhodesiense*	JE15	Serere, Uganda	2003	Human	+	+	−	+	+	+
*T. b. rhodesiense*	EATRO 149	Nyanza, Kenya	1961	Human	+	−	−	+	+	+
*T. b. rhodesiense*	KETRI 2473	Nyanza, Kenya	1970	Human	+	+	−	+	+	+
*T. b. rhodesiense*	EATRO 2636	Mozambique	1983	Human	+	−	−	+	+	+
*T. b. rhodesiense*	KETRI 3537	Bugoma, Kenya	1998	Human	+	+	−	+	+	+
*T. b. rhodesiense*	KETRI 3624	Busia, Kenya	1998	Human	+	+	−	+	+	+
*T. b. rhodesiense*	KETRI 3639	Busia, Kenya	1999	Human	+	+	−	+	+	+
*T. b. rhodesiense*	TMRS 51a	Kibondo, Tanzania	2004	Human	+	+	−	+	+	+
*T. b. rhodesiense*	TMRS 51b	Kibondo, Tanzania	2004	Human	+	+	−	+	+	+
*T. b. rhodesiense*	TMRS 51c	Kibondo	2005	Human	+	+	−	+	+	+
*T. b. rhodesiense*	TMRS 52a	Urambo, Tanzania	2005	Human	+	+	−	+	+	+
*T. b. rhodesiense*	TMRS 52b	Urambo, Tanzania	2004	Human	−	−	−	+	+	+
*T. b. rhodesiense*	TMRS 52c	Urambo, Tanzania	2006	Human	−	−	−	+	+	+
*T. b. rhodesiense*	TMRS 53a	Mpanda, Tanzania	2005	Human	+	−	−	+	+	+
*T. b. rhodesiense*	TMRS 53b	Mpanda, Tanzania	2005	Human	−	−	−	+	+	+
*T. b. rhodesiense*	TMRS 53c	Mpanda, Tanzania	2005	Human	+	+	−	+	+	+
*T. b. rhodesiense*	TMRS JM	Kasulu, Tanzania	2001	Human	+	−	−	+	+	+
*T. b. rhodesiense*	TMRS 58	Mpanda, Tanzania	2006	Human	+	+	−	+	+	+
*T. b. rhodesiense*	TMRS 4M	Urambo, Tanzania	2006	Human	−	−	−	+	+	+
*T. b. gambiense*	MOS^d^	Mbam, Cameroon	1974	Human	+	−	+	−	−	−
*T. b. gambiense*	PT16^d^	Ivory Coast	1992	Human	+	−	+	−	−	−
*T. b. gambiense*	Boula^d^	Bouenza, Congo	1989	Human	+	−	+	−	−	−
*T. b. gambiense*	NW2^d^	Uganda	1992	Human	+	−	+	−	−	−
*T. b. gambiense*	Dal 972^d^	Daloa, Ivory Coast	1978	Human	+	−	+	−	−	−
*T. b. brucei*	LUMP 266^d^	Kiboko, Kenya	1969	Fly, *G. pallidipes*	+	−	−	−	−	−
*T. b. brucei*	KP2N^d^	Kouassi-Perita, Ivory Coast	1982	Fly, *G. palpalis*	+	−	−	−	−	−
*T. b. brucei*	B8/18^d^	Nsukka, Nigeria	1962	Pig	+	−	−	−	−	−
*T. b. brucei*	J10^d^	Luangwa Valley, Zambia	1973	Hyena	+	−	−	−	−	−
*T. b. brucei*	STIB 215^d^	Serengeti, Tanzania	1971	Lion	+	−	−	−	−	−
*T. b. brucei*	Katerema^d^	Uganda	1990	Cow	+	−	−	−	−	−
*T. evansi*	SA17	Isiolo, Kenya	2003	Camel	+	−	−	−	−	−
*T. evansi*	KETRI 2426	Ukunda, Kenya	1978	Camel	+	−	−	−	−	−
*T. evansi*	KETRI 3093	Colombia, South America	1979	Horse	+	−	−	−	−	−
*T. evansi*	SA263	Samburu, Kenya	2003	Camel	+	−	−	−	−	−
*T. evansi*	KETRI 243	Kulal, Kenya	1979	Camel	+	−	−	−	−	−
*T. evansi*	KETRI 3565	Athi River, Kenya	1994	Camel	+	−	−	−	−	−
*T. congolense* forest	Cam 22^d^	Mbetta, Cameroon	1984	Goat	−	−	−	−	−	−
*T. c.* kilifi	WG5^d^	Kenya	1980	Sheep	−	−	−	−	−	−
*T. c.* savannah	KETRI 1869	Kenya	—	—	−	−	−	−	−	−
*T. simiae*	Ken 4^d^	Keneba, The Gambia	1988	Fly	−	−	−	−	−	−
*T. simiae* tsavo	KETRI 1864^d^	Kenya	—	Fly	−	−	−	−	−	−
*T. godfreyi*	Ken 7^d^	Kenya	1988	Fly, *G. morsitans*	−	−	−	−	−	−
*T. vivax*	Y58	Nigeria	—	—	−	−	−	−	−	−

JE samples are from Uganda and were processed using Sigma Genomic DNA extraction kit, followed by precipitation with 3 M sodium acetate and absolute alcohol. TMRS samples were from Tanzania and the DNA was prepared using Qiagen DNA extraction kit.

a Subgenus *Trypanozoon* PCR test [7].

b *T. b. rhodesiense* PCR test [3].

c *T. b. gambiense* PCR test [17].

d Source: Wendy Gibson, University of Bristol, UK.

e The samples were processed using the saponin lysis method [5].

f DNA prepared directly from human blood.

g DNA prepared directly from human CSF.

h Source: Philippe Büscher, Institute of Tropical Medicine Antwerp, Belgium. DNA prepared with Qiagen DNA extraction kit.

nd, not done in this study (identification reported [17]).

### Polymerase chain reaction

Trypanosomes belonging to the subgenus *Trypanozoon* were analysed using TBR1 and 2 primers [7]. Furthermore *T. b. rhodesiense* was detected by a PCR specific for the *SRA* gene [3]), whereas *T. b. gambiense* was detected using a PCR for the *T. b. gambiense*-*specific glycoprotein (TgsGP)* gene [17].

### LAMP reaction

LAMP reactions of 25 µl were standardised for optimal reagent concentrations, temperature, and time conditions using *T. b. rhodesiense* isolate LVH 56 and following the Taguchi design [18]. Briefly, the FIP and BIP were varied from 0.8 µM to 2.4 µM, dNTPs from 100 µM to 400 µM, betaine from 0.2 M to 0.8 M, and MgSO_4_ from 0 to 4 mM. The FIP, BIP, F3, and B3 primers were designed using the PrimerExplorer v3 software (http:/primerexplorer.jp/lamp) based on the *SRA* gene sequence (GenBank accession number Z37159) (Table 2). Loop primers [loop forward (LF) and loop backward (LB)] were designed manually. The reactions were optimised at 2.0 µM for FIP and BIP primers, 0.8 µM for loop primer (LF and LB), 0.2 µM for F3 and B3 outer primers, 200 µM for each dNTP, 0.8 M betaine (Sigma), 20 mM Tris-HCl (pH 8.8), 10 mM KCl, 10 mM (NH_4_)_2_SO_4_, 2 mM MgSO_4_, 0.1% Triton X-100, and 8 U of *Bst* DNA polymerase large fragment (New England Biolabs). For real-time reactions 3.34 µM SYTO-9 fluorescence dye (Molecular Probes) was added. The template was ∼100 pg for trypanosome lysate DNA samples and 2 µl of buffy coat and supernatant prepared from boiled blood. To find the optimum temperature for the LAMP test, the reactions were carried out for 1 h at 58, 60, 62, and 64°C using the Rotor-Gene 3000 thermocycler (Corbett Research) or in a water bath at the same temperature settings. The reaction was terminated by increasing the temperature to 80°C for 4 min.

**Table 2:** Nucleotide Sequences for the *SRA* LAMP Primers

Primer	Type	Sequence^a^ (5′–3′)	Length	Amplicon Size^b^	Target
*SRA*-F3	F3	GCGGAAGCAAGAATGACC	18	162	*SRA* gene
*SRA*-B3	B3	TCTTACCTTGTGACGCCTG	19	—	—
*SRA*-FIP	FIP (F1c+F2)	GGACTGCGTTGAGTACGCATCCGCAAGCACAGACCACAGC	40	—	—
*SRA*-BIP	BIP (B1c+B2)	CGCTCTTACAAGTCTTGCGCCCTTCTGAGATGTGCCCACTG	41	—	—
*SRA*-LF	LF	CGCGGCATAAAGCGCTGAG	19	—	—
*SRA*-LB	LB	GCAGCGACCAACGGAGCC	18	—	—

a Accession number Z37159.

b The length between F2 and B2 is 162 bp. However, the amplified amplicon sizes will be more than 162 bp since the FIP and BIP primers consist of F1c (21 bp) and B1c (21 bp), respectively.

### Detection of LAMP product

Three methods were used to analyse DNA amplification, and included electrophoresis in 1.5% agarose gels stained with ethidium bromide, direct visual inspection of the LAMP product after addition of 1 µl of 1/10 dilution of SYBR Green I (Invitrogen), and by monitoring fluorescence of the double-stranded DNA (dsDNA)-specific dye SYTO-9 [19] in a Rotor-Gene 3000 thermocycler. Real-time fluorescence data was obtained on the FAM channel (excitation at 470 nm and detection at 510 nm) [19]. Three approaches were used to confirm that the *SRA* LAMP test amplified the correct target: (1) the product was digested with restriction enzyme *Rsa*I (New England Biolabs) at 37°C for 3 h, followed by electrophoresis in 3% agarose gel; (2) following amplification, the DNA melting curves were acquired on the FAM channel using 1°C steps, with a hold of 30 s, from 62 to 96°C [19]; and (3) some of the LAMP amplicon bands were excised from an agarose gel and cloned into a TOPO-TA vector (Invitrogen), transformed in *E. coli* and inserts sequenced using an automated DNA 3730 analyser (Applied Biosystems). The resulting sequences were aligned with the target sequence using the DNAman computer software version 5.0 (Lynnon Biosoft).

### Sensitivity and specificity of LAMP

10-fold dilutions were made from infected mouse blood containing 1.0×10^6^ trypanosomes/ml and from 100 ng of purified *T. b. rhodesiense* DNA, and used to determine the analytical sensitivity of *SRA* LAMP and PCR tests. The reactions were done in triplicates and repeated after 2 wks. The LAMP test was carried out using both cold and heated templates. The specificity of the tests were assessed with DNA from human, tsetse fly, bovine, *Plasmodium falciparum*, and trypanosomes belonging to other species (*Trypanosoma brucei brucei*, *T. b. gambiense, T. b. evansi, Trypanosoma congolense* savannah, *T. c.* kilifi, *T. c*. forest, *Trypanosoma simiae, T. s.* tsavo, *Trypanosoma godfreyi, Trypanosoma vivax,* and *Trypanosoma lewisi*).

## Results

### LAMP test

**Figure 1:**
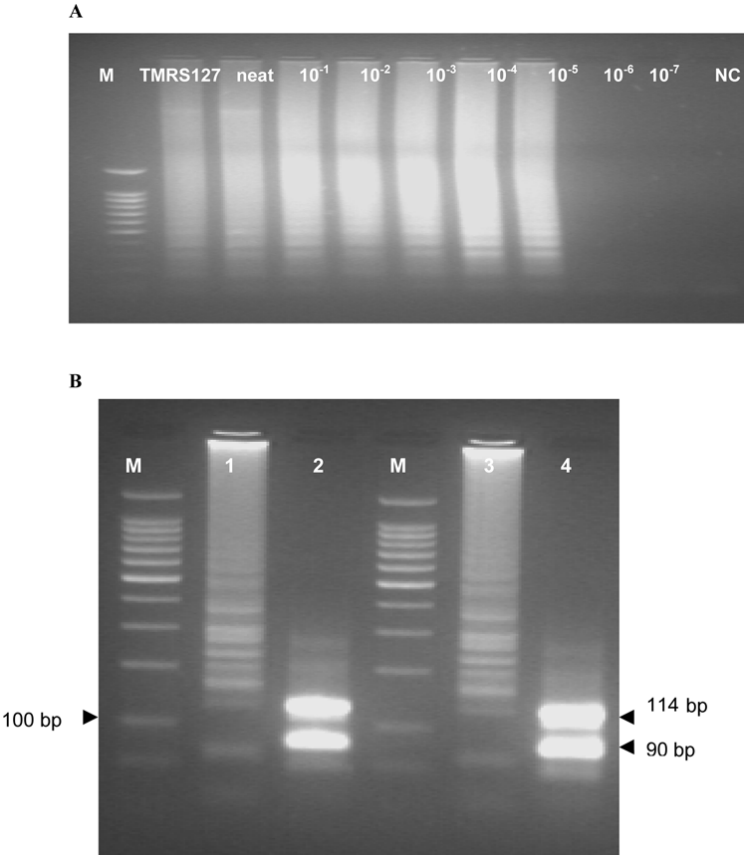
Analytical Sensitivity of *SRA* LAMP and Restriction Enzyme Digestion Results. (A) Sensitivity of the *SRA* LAMP assay using DNA lysate from *T. b. rhodesiense* isolate LVH 56. Sensitivity results for 10-fold dilutions from infected mouse blood showed identical results. The lysate/supernatant was heated at 96°C for 1 min before being added to the reaction mixture. The reactions which were done in triplicates showed detection limit of ≈1 pg an equivalent of dilution 10^−5^. M, 100 bp marker, TMRS 127, *T. b. rhodesiense*, neat (100 ng), 10^−1^ (10 ng), 10^−2^ (1 ng), 10^−3^ (100 pg), 10^−4^ (10 pg), 10^−5^ (1 pg), 10^−6^ (100 fg), 10^−7^ (10 fg), and NC, negative control. The detection limit for *SRA* PCR was 10^−2^ (100 pg≈1,000 trypanosomes/ml). (B) Electrophoresis results for isolates TMRS 127 (lane 1) and LVH 56 (lane 3), and their *Rsa*I restriction enzyme digests (lane 2 for TMRS 127 and lane 4 for LVH 56). M, 100 bp marker.

**Figure 2 pntd-0000147-g002:**
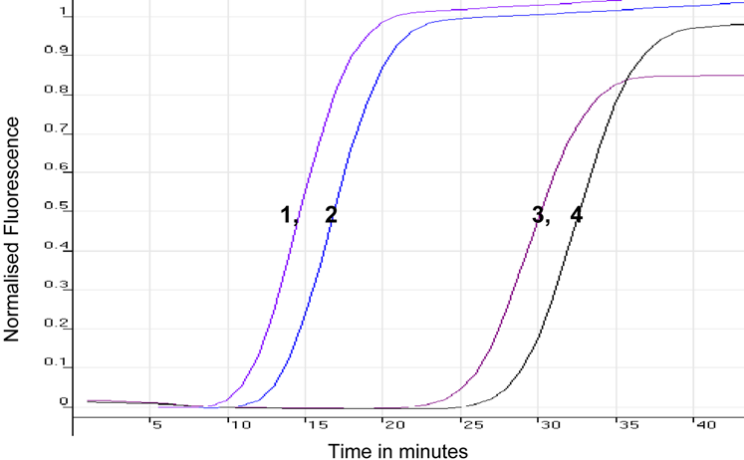
Comparison of the Sensitivity of *SRA* LAMP Under Different Conditions. Sensitivity was compared using preheated and cold template for *T. b. rhodesiense* isolate LVH 56 (1 and 3) and 058 (2 and 4), respectively, and as monitored using the Rotor-Gene 3000 thermocycler. Preheating of the template accelerates the detection of a positive reaction by approximately 12 min.

**Figure 3 pntd-0000147-g003:**
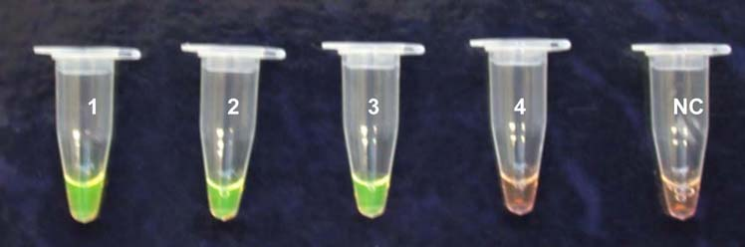
Visual Appearance of Postassay *SRA* LAMP Reactions from Isolates after the Addition of 1 µl of 1/10 Dilution of SYBR Green I. Positive samples produce a green colour almost immediately (tubes 1, 2, and 3 containing 100 pg of *T. b. rhodesiense* DNA) while negatives remain orange (tube 4 and NC, negative control).

**Figure 4:**
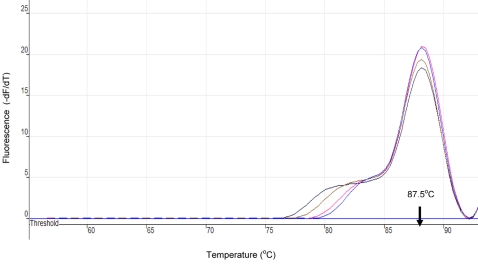
Melting Curves for *T. b. rhodesiense SRA* LAMP Product as Monitored in Rotor-Gene 3000. The curves were obtained after LAMP amplification for 35 min and detected on the FAM channel using 1°C steps, and a hold of 30 sec, at each step from 60 to 96°C. All isolates had a melting temperature (T_m_) of ∼87.5°C indicating similar sequences, and hence similar amplicon.

The results of the *SRA* LAMP assay are shown in Figures 1–4 and Table 1. When the test was carried out without loop primers a product was detected after 50 min. The inclusion of loop primers reduced the reaction time from an average of 50 min down to between 20 and 25 min and increased the sensitivity 100-fold. The best results were obtained when the reaction temperature was maintained at 62°C. All the positive LAMP reactions produced a characteristic ladder of multiple bands on an agarose gel (Figure 1A and 1B), indicating that stem-loop DNA with inverted repeats of the target sequence was produced. Positive reactions turned green on addition of SYBR Green I, while the negative ones remained orange (Figure 3). *Rsa*I restriction enzyme digestion and electrophoresis gave the predicted sizes of 90 bp and 114 bp (Figure 1B). The *SRA* LAMP amplicons showed reproducible melt curves with a T_m_ of ∼87.5°C, suggesting amplicons of the same sequence (Figure 4). The cloned sequence showed 100% identity with the target sequence, and revealed that the length varied with sequence repeats of primers and there complementary sequences. The analytical sensitivity of *SRA* LAMP assay improved from a dilution of 10^−4^ to 10^−6^ when a template (DNA or supernatant) was preheated before being added to a reaction (Figure 2), with the best detection limit of dilution 10^−7^ recorded with supernatant prepared from the buffy coat. The classical PCR based on the same gene [3] showed a detection limit of dilution 10^−4^. The *SRA* LAMP detected all the 49 (100%) *T. b. rhodesiense* (including the six samples isolated directly from HAT patients), while TBR1 and 2 primers detected 39 out of 46 (84.8%) and *SRA* PCR detected 31 out of 46 (67.4%) samples (Table 1). The *SRA* LAMP test was specific and no cross-reaction was recorded with nontarget DNA.

## Discussion

In the present study we were able to demonstrate the successful amplification of *T. b. rhodesiense* DNA within 20–25 min at 62°C using the *SRA* LAMP assay. However, we set the optimal time at 35 min to amplify DNA at low concentrations. The results of the *SRA* LAMP assay were identical when either a water bath or a thermocycler was used to maintain the temperature at 62°C, demonstrating its robustness. Preheating of the template increased the efficiency of the assay by shortening the duration (Figure 2) and increasing sensitivity of the test. DNA amplification is preceded by strand separation under isothermal conditions using betaine, which destabilises the DNA helix [8]. It would appear that preheating of the sample produced a faster and/or a greater amount of strand separation, which translated into a far more rapid assay. All positive samples detected by gel electrophoresis or in real-time using SYTO-9 fluorescence dye could also be detected visually by addition of SYBR Green I to the product. This ability highlights another advantage of LAMP technique: the results of amplification can visually be observed through addition of a DNA intercalating dye (Figure 3), eliminating the need for gel electrophoresis and greatly reducing the time taken for result analysis.

When pure trypanosome DNA was used, the detection limit of the *SRA* LAMP test without loop primers was an equivalent of 1,000 trypanosomes/ml. This limit was improved to an equivalent of one trypanosome/ml with the inclusion of loop primers. Increased sensitivity and reduction in LAMP reaction time with the addition of loop primers is well documented [20] and has been demonstrated in detection of *Mycobacterium* [11], periodontal pathogens [12], and *Plasmodium falciparum* malaria [10]. Loop primers accelerate the LAMP reaction by hybridising to the stem-loop region, initiating further DNA amplification [20]. When different templates were used, heat-treated buffy coat from mice blood performed better than the supernatant obtained after boiling blood samples. The higher sensitivity recorded could be the effect of concentrating the parasites in the buffy coat through centrifugation; therefore, buffy coat seems a superior template for *SRA* LAMP test.

The robustness of the LAMP test is demonstrated by the ability to amplify target DNA from various templates without the expensive and time-consuming process of DNA purification. We observed no inhibitory effects in using 2–5 µl of supernatant in a 25 µl reaction or an increase in sensitivity beyond 2 µl, indicating that this volume was the optimal for our samples. The possibility of using heat-processed samples without compromising sensitivity eliminates the need for DNA extraction and further shortens the LAMP reaction. Other studies have shown superior tolerance of LAMP tests for biological substances [9],[13] and heat processed blood has been used successfully in detection Malaria [10]. The method of template preparation for use in LAMP tests, however, needs to be further developed.

The potential usefulness of *SRA* LAMP is confirmed by its ability to detect *T. b. rhodesiense* directly from parasitaemic and apparently aparasitaemic clinical samples (human blood and CSF). The human blood (JE2 and JE3) and CSF samples JE8–JE10 used in the present study were negative by microscopy at the time of sampling. Parasites were demonstrated only following inoculation of the samples in mice. When the samples were tested, they were positive by *SRA* LAMP assay while only JE4, JE9, and JE10 were positive using TBR PCR (Table 1) [7]. Detection of aparasitaemic samples demonstrates one of the practical values of *SRA* LAMP in sleeping sickness diagnosis-time-consuming parasite multiplication assays in mice are unnecessary, and early diagnosis increases the chances of cure after treatment.

In the present study, amplification of the target sequence was confirmed by restriction enzyme digestion using *Rsa*I, melting curve analysis, and sequence analysis. It is important to distinguish *T b. rhodesiense* and *T. b. gambiense* since the two parasitic infections have different treatments. In recent years the *T. b. rhodesiense* region in Southern Uganda has been expanding towards the *T. b. gambiense* focus as a result of livestock movement [21],[22]. There is therefore a need to continue development of rapid and sensitive techniques to differentiate the two parasites and to compliment the available PCR tests, and to this end the *SRA* LAMP assay has shown great potential for this application.

The LAMP test should theoretically not amplify nontarget sequences, since the specificity is enhanced by using a set of six primers. However there is a high risk of amplicon contamination since the tubes have to be opened to add the dye. Analysis of any false positive reactions through sequencing and restriction enzyme analysis would easily distinguish between false positive and contamination. To reduce the chances of contamination, similar protocols to those followed for PCR are required. However, the great potential for LAMP is that reactions can be performed and results read without opening tubes [23]. On this end, more work is needed to develop such a closed reaction system for diagnosing sleeping sickness.

This study has shown that the *SRA* LAMP assay could be developed into an assay for *T. b. rhodesiense* that is simple to use at point of care. The detection of the equivalent of one trypanosome/ml in the buffy coat (with the possibility of reducing this further to 0.1 trypanosomes/ml) compares well with the normal parasitaemia in humans. Since DNA amplification and reading of results require minimum equipment, the technique has great potential for use in the HAT-endemic countries as back-up test for other HAT tests currently in use.
